# Statins inhibit insulin-like growth factor action in first trimester placenta by altering insulin-like growth factor 1 receptor glycosylation

**DOI:** 10.1093/molehr/gau093

**Published:** 2014-10-09

**Authors:** Karen Forbes, Vinit K. Shah, Kirk Siddals, J. Martin Gibson, John D. Aplin, Melissa Westwood

**Affiliations:** 1 Maternal and FetalHealth Research Centre, University of Manchester, Manchester Academic Health Sciences Centre, Manchester M13 9WL, UK; 2 Maternal and Fetal Health Research Centre, St. Mary's Hospital, Central Manchester University Hospitals NHS Foundation Trust, Manchester Academic Health Science Centre, Manchester M13 9WL, UK; 3 Centre for Imaging Sciences, Institute of Population Health, University of Manchester, Manchester Academic Health Sciences Centre, Manchester M13 9PY, UK

**Keywords:** IGF, glycosylation, proliferation, trophoblast, HMG CoA reductase

## Abstract

The rapid rise in obesity, metabolic syndrome and type 2 diabetes is one of the major healthcare problems of the Western world. Affected individuals are often treated with statins (3-hydroxy-3-methylglutaryl co-enzyme A [HMG CoA] reductase inhibitors) to reduce circulating cholesterol levels and the risk of developing cardiovascular disease; given the evolving demographic profile of these conditions, such drugs are increasingly prescribed to women of reproductive age. We have previously shown that exposure of placental tissue to statins inhibits the action of insulin-like growth factors (IGF)-I and -II which are key regulators of trophoblast proliferation and placental development. N-linked glycans in the IGF receptor, IGF1R, influence its presentation at the cell surface. This study aimed to determine whether statins, which are known to affect N-glycosylation, modulate IGF1R function in placenta. Treatment of first trimester villous tissue explants with statins (pravastatin or cerivastatin) or inhibitors of N-glycosylation (tunicamycin, deoxymannojirimycin or castanospermine) altered receptor distribution in trophoblast and attenuated proliferation induced by IGF-I or IGF-II (Ki67; *P* < 0.05, *n* = 5). Decreased binding of Phaseolus vulgaris lectin and phytohaemagglutinin to IGF1R immunoprecipitated from treated explants demonstrated reduced levels of complex N-linked glycans. Co-incubation of tissue explants with statins and farnesyl pyrophosphate (which increases the supply of dolichol intermediates), prevented statin-mediated disruption of IGF1R localization and reversed the negative effect on IGF-mediated trophoblast proliferation. These data suggest that statins attenuate IGF actions in the placenta by inhibiting N-linked glycosylation and subsequent expression of mature IGF1R at the placental cell surface.

## Introduction

The rapid rise in obesity, metabolic syndrome and type 2 diabetes is one of the major healthcare problems of the Western world (Wild *et al.*, 2004; Shaw *et al.*, 2010). Affected individuals are often treated with 3-hydroxy-3-methylglutaryl co-enzyme A (HMG CoA) reductase inhibitors (statins) to reduce circulating cholesterol levels and the risk of developing cardiovascular disease. Given that the incidence of obesity and diabetes has risen amongst younger populations (Flegal *et al.*, 1998; Ogden *et al.*, 2014) and that many women are delaying child bearing (Heffner, 2004), statins are increasingly prescribed to women of reproductive age.

Studies in rodents suggest that administration of statins may have beneficial effects in pregnancy complications, such as pre-eclampsia, that are associated with altered vascular function (Fox *et al.*, 2011; Kumasawa *et al.*, 2011; Bauer *et al.*, 2013). These findings have led to a surge in the number of studies investigating the use of statins in human pregnancy and two randomized control trials are underway (ISRCTN, 2009; Costantine and Cleary, 2013). However, statins can affect placental development (Kenis *et al.*, 2005; Tartakover-Matalon *et al.*, 2007; Forbes *et al.*, 2008a; Odiari *et al.*, 2012) and cross the placental barrier to target the developing fetus, so their use in pregnancy is still contraindicated (Godfrey *et al.*, 2012; Lecarpentier *et al.*, 2012; Winterfeld *et al.*, 2013). Nonetheless, given that ∼50% of pregnancies are unplanned (Finer and Henshaw, 2006), it is likely that in the coming years, inadvertently or otherwise, many pregnant women and their fetuses will be exposed to statins.

In normal placental development, cytotrophoblast progenitor cells proliferate and differentiate into one of two subtypes; syncytiotrophoblast, which is in direct contact with maternal blood and responsible for nutrient and gas exchange (Aplin, 2010); or extravillous trophoblast which migrate into the maternal uterine decidua and myometrium (Aplin, 1991). The syncytium is terminally differentiated, thus growth and then maintenance of the syncytial surface area occur via the continuous proliferation, differentiation and fusion of underlying cytotrophoblast cells (reviewed in Aplin, 2000). Altered rates of cytotrophoblast proliferation are associated with different pathologies; enhanced levels are associated with increased fetal growth (macrosomia) whilst low levels are linked to reduced (FGR) fetal growth (Jansson and Powell, 2006).

The insulin-like growth factor (IGF) axis is an important regulator of placental cell turnover and fetal growth (Forbes and Westwood, 2008). IGF-I and -II exert their actions in the placenta by binding to the type-1 IGF receptor (IGF1R) in trophoblast, activating the MAPK and PI3K pathways and initiating a cascade of phosphorylation events to promote mitogenesis and protect against apoptosis (Forbes *et al.*, 2008b). Altered IGF signalling results in aberrant placental growth (Walenkamp *et al.*, 2006; Kruis *et al.*, 2010). We have reported that treatment of first trimester human placental tissue with statins (pravastatin or cerivastatin) decreases IGF-mediated proliferation (Forbes *et al.*, 2008a). The actions of statins are not limited to modulation of cholesterol levels; they cause depletion of mevalonate, and consequently, farnesyl pyrophosphate, resulting in reduced levels of dolichol phosphate, an essential cofactor for protein *N*-linked glycosylation. Co-translational N-glycosylation influences protein folding and its inhibition can lead to catabolism of unfolded proteins and depletion of functional proteins from their sites of action, including the cell surface. IGF1R contains several N-glycans (Siddals *et al.*, 2004, 2011), and inhibition of N-glycosylation alters cell surface abundance and blocks IGF action in tumour cells (Contessa *et al.*, 2008) and vascular smooth muscle cells (Siddals *et al.*, 2011). Plant lectins, carbohydrate-binding proteins with a range of sugar specificities that include various types of N-linked oligosaccharide, have been used extensively in histochemical studies to delineate the diversity of glycans at the maternal–fetal interface (Jones *et al.*, 2007), and introduce the hypothesis of a ‘glycocode’ that defines interfacial characteristics and influences cellular interactions (Jones and Aplin, 2009; Aplin and Jones, 2012). This study explored the hypothesis that statins attenuate IGF-induced proliferation in the placenta by altering N-glycosylation and cell surface expression of IGF1R.

## Materials and Methods

### Materials

HMG-CoA reductase inhibitors were a kind gift of Bayer Pharmaceuticals PLC and AstraZeneca PLC. Deoxymannojirimycin and tunicamycin were purchased from Calbiochem. Farnesyl pyrophosphate and castanospermine were purchased from Sigma.

### Tissue culture

Late first trimester (8–12 week) human placental tissue was collected with maternal informed consent and approval from our Local Research Ethics Committee following elective surgical or medical termination of pregnancy. Tissue was transferred into a 1:1 mixture of serum-free Dulbecco's Modified Eagle's Medium (DMEM) and Ham's F12 (F12) containing 100-units/ml penicillin, 100 μg/ml streptomycin and 2 mM l-glutamine (DMEM/F12), dissected into 5 mm^3^ pieces under sterile conditions and immediately used in the experiments described below.

### HMG-CoA reductase inhibitor and glycosylation inhibitor treatments

Tissue (*n* = 5) was pre-incubated with cerivastatin (50 μnM), pravastatin (250 μnM) or the glycosylation inhibitors: tunicamycin (1 μg/ml; an inhibitor of N-acetylglucosamine transferase which prevents formation of dolichyl pyrophospho-N-acetylglucosamine, blocking N-glycosylation of newly synthesized proteins (McDowell and Schwarz, 1988)), castanospermine (5 μg/ml; a glucosidase inhibitor that prevents exit of nascent glycoprotein from the ER) or deoxymannojirimycin (DMJ, 0.5 mM; a mannosidase inhibitor, which prevents the conversion of high mannose type to complex type oligosaccharides (Fuhrmann *et al.*, 1984)), in DMEM/F12 for 24 h. IGF-I (10 μnM) or IGF-II (10 μnM) was then added and explants were cultured for a further 24 h. In some experiments (*n* = 5), 20 μM farnesyl pyrophosphate (FPP)—a concentration that we have previously shown reverses the effect of cerivastatin in 3T3L1 cells (Siddals *et al.*, 2004)—was added to explants at the same time as HMG-CoA reductase inhibitors.

### Immunohistochemistry

Following exposure to inhibitors and/or IGF, placental tissue was fixed in 4 w/v % paraformaldehyde overnight, embedded in paraffin wax and sectioned (5 µM). Sections were boiled in 0.1 μM sodium citrate buffer to maximize antigen retrieval and then incubated with rabbit anti-IGF1Rβ (1:100; Biosource, UK), mouse anti-Ki67 (MIB-1 clone, 1:200; DakoCytomation Ltd, Cambridgeshire, UK), non-immune rabbit IgG (Sigma, UK) or non-immune mouse IgG (DakoCytomation Ltd, Cambridgeshire, UK) followed by biotinylated swine anti-rabbit IgG antibody or biotinylated goat anti-mouse IgG (1:200; DakoCytomation Ltd, Cambridgeshire, UK). Staining was visualized using the avidin-peroxidase method with haematoxylin counterstain as previously described (Forbes *et al.*, 2008b) and images were captured using a Leica microscope. Levels of cytotrophoblast proliferation were then determined as previously described (Forbes *et al.*, 2008b) and expressed as a percentage of total cytotrophoblast number. Comparisons between groups were made using the Kruskal–Wallis test followed by a Dunn's *post hoc* test. Data were considered significant at *P* < 0.05.

### Western blotting

IGF receptor processing was assessed by immunoblotting. Lysates of whole placental tissue were prepared in RIPA buffer as previously described (Forbes *et al.*, 2009). One hundred μg protein from each sample was resolved by SDS–PAGE and transferred to nitrocellulose membranes for western blotting with antiserum specific for IGF1Rβ (rabbit polyclonal; 1:500; Santa Cruz Biotechnology, Inc., CA, USA). Immune complexes were visualized by probing with HRP-anti-rabbit-IgG followed by enhanced chemiluminescence (ECL).

### Analysis of IGF1R glycosylation

#### Immunoprecipitation of IGF1R

Lysates of placental explants treated with HMG co-reductase or glycosylation inhibitors (*n* = 3) were pre-cleared with protein-G-Sepharose, then incubated with anti-IGF1Rβ antibody (mouse monoclonal IgG, Santa Cruz Biotechnology) and protein-G-Sepharose overnight at 4°C. The immune complexes were pelleted by centrifugation, washed three times with ice-cold phosphate-buffered saline and then resuspended in reducing SDS loading buffer (0.125 M Tris HCl, pH 6.8, 2 w/v % SDS, 10 v/v % glycerol, 5 v/v % 2-mercaptoethanol, 0.25 v/v % bromphenol blue). IGF1R enrichment was confirmed by western blot analysis of immunoprecipitates as described above.

#### Lectin dot blots

IGF1R glycosylation status was determined by dot blot using a modification to a previously published method (Schumacher *et al.*, 1992) and two lectins, with different sugar specificities (Table I), that have previously been shown to recognize IGF1R (Masnikosa *et al.*, 2006). Briefly, 10 μg of each sample (*n* = 3) was applied to nitrocellulose membranes. Membranes were dried at room temperature for 15 min and non-specific binding sites blocked by soaking in 5 w/v % BSA for 30 min at room temperature. Membranes were incubated with biotin-labelled lectins: Phaseolus vulgaris lectin (ePHA) or l-phytohaemagglutinin (lPHA) (10 μg/ml in 0.1 M Tris-buffered saline (TBS)) for 1 h at room temperature, and then washed three times (10 min in TBS containing 0.2 v/v % Tween 20) before incubation with HRP-conjugated streptavidin (1:2000; Cell Signaling Technologies, UK) for 1 h. Binding was visualized by ECL and intensity of dots quantified by densitometry using Image J software.

**Table I:** Lectins used for dot blots.

Lectin	Abbreviation	Gylcosylation sites detected
*Phaseolus vulgaris* lectin	E-PHA	Bisected complex-type N-glycans; does not bind any known glycolipid or O-glycan (Jones *et al*., 2008)
Phytohaemagglutinin	L-PHA	Certain branched, complex-type N-glycans containing the pentasaccharide sequence Galβ1-4GlcNAcβ1-2(Galβ1-4GlcNAcβ1-6)Manα1-R. Does not bind to any known glycolipid or O-glycan. (Jones *et al*., 2008)

## Results

### Glycosylation inhibitors attenuate IGF-induced proliferation in a manner similar to HMG-CoA reductase inhibitors

**Figure 1:**
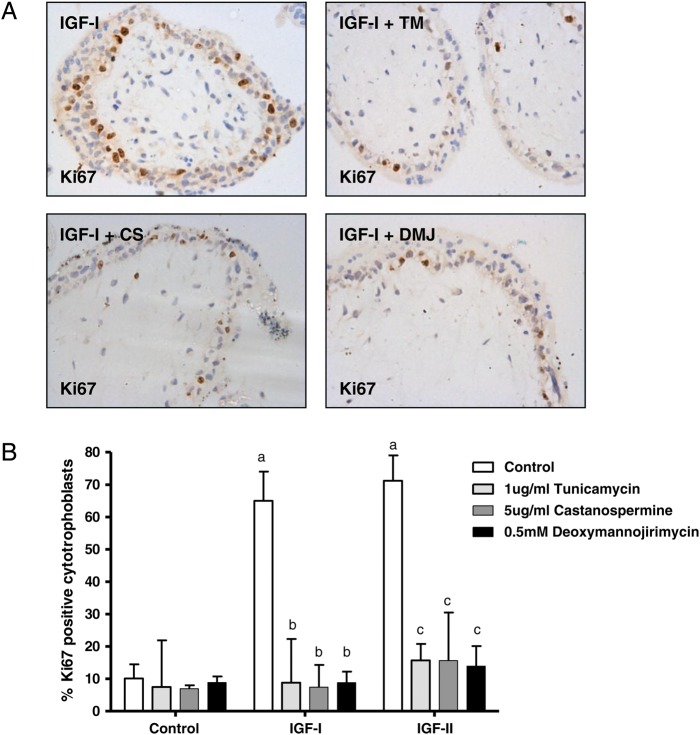
Glycosylation inhibitors attenuate insulin-like growth factor (IGF)-induced proliferation. Explants of human first trimester placenta (*n* = 5) were incubated in the absence or presence of tunicamycin (TM; 1 μg/ml), castanospermine (CS; 5 μg/ml) or deoxymannojirimycin (DMJ; 0.5 mM) for 24 h before the addition of vehicle, IGF-I (10 nM) or IGF-II (10 nM) and culture for a further 24 h. Proliferation was assessed by using immunohistochemistry (**A**) to determine the number of Ki67-positive cytotrophoblast as a percentage of total cytotrophoblast and data are presented (**B**) as median and range of five independent experiments. a: <0.05 versus control untreated, b: <0.05 versus IGF-I alone, c: <0.05 versus IGF-II alone.

The importance of IGF1R glycosylation for IGF-induced cytotrophoblast proliferation was investigated by examining the effect of treatment with inhibitors of N-linked glycosylation. First trimester placental explants were pre-treated with inhibitor for 24 h before exposure to IGF-I (10 nM) or IGF-II (10 nM) for a further 24 h. Each of the inhibitors attenuated IGF-stimulated trophoblast proliferation by at least 6-fold (Fig. 1). This degree of reduction in IGF-induced proliferation was comparable to the effect observed following treatment with HMG-CoA reductase inhibitors (at least 3-fold; see Fig. 4 and data within Forbes *et al.*, 2008b) suggesting that the ability of statins to attenuate IGF-actions in the first trimester placenta may be a consequence of altered IGF1R glycosylation.

### Placental IGF1R glycosylation and cell surface localization are altered by HMG-CoA reductase inhibitors in the first trimester human placenta

**Figure 2:**
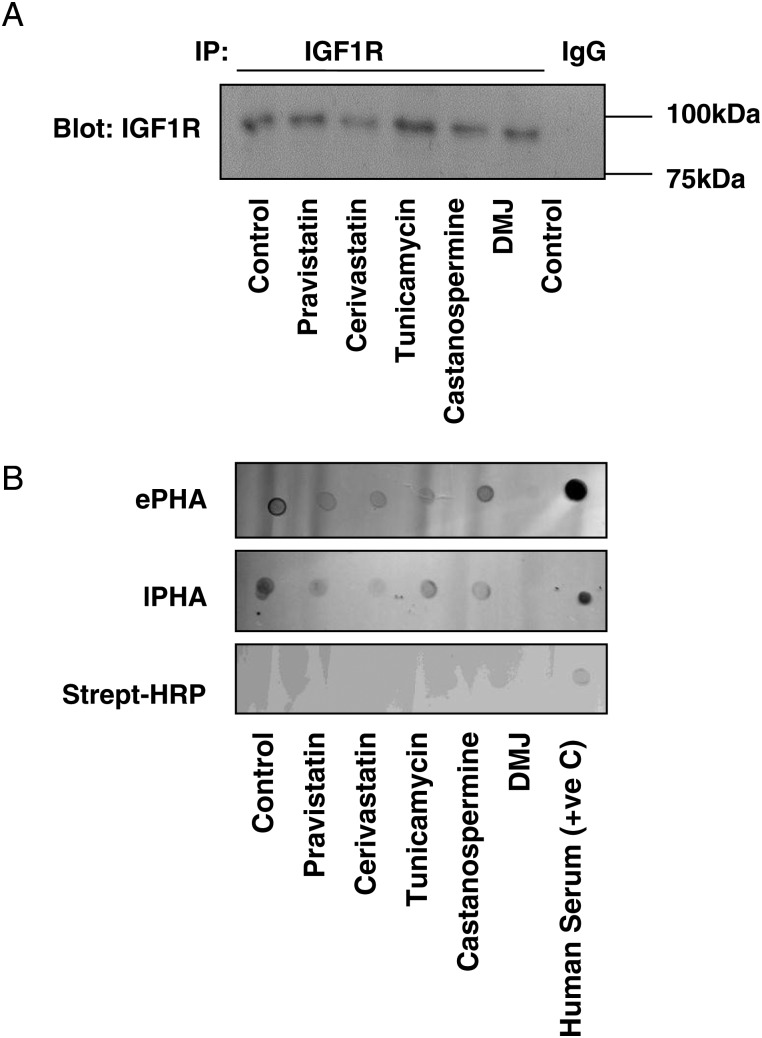
Insulin-like growth factor 1 receptor (IGF1R) glycosylation in the human placenta is reduced by 3-hydroxy-3-methylglutaryl co-enzyme A [HMG-CoA] reductase inhibitors. First trimester placental explants were incubated in the absence or presence of pravastatin (250 nM), cerivastatin (50 nM), tunicamycin (1 μg/ml), castanospermine (5 μg/ml) or deoxymannojirimycin (DMJ; 0.5 mM) for 24 h. IGF1R was immunoprecipitated from the samples using IGF1R specific antibodies (or mouse IgG as a negative control) (**A**) and its glycosylation status analysed by lectin dot blot (**B**) using lectins that recognize different glycosylation sites: Phaseolus vulgaris lectin (ePHA) and l-phytohaemagglutinin (lPHA). Both statin treatment and glycosylation inhibitors reduced the levels of IGF1R glycosylation detected by both ePHA and lPHA. Human serum was used as a positive control and specificity of the detection method was confirmed by the absence of positive signal when no lectins were used (streptavidin-horse radish peroxidase [HRP] only). Each image is representative of experiments performed on three individual placentas.

The effects of pravastatin, cerivastatin and the three inhibitors of glycosylation on the glycosylation of placental IGF1R were examined by assessing the binding of two lectins that recognize N-linked glycans: Phaseolus vulgaris lectin (ePHA) and l-phytohaemagglutinin (lPHA). Lysates from placental explants were enriched for IGF1R by immunoprecipitation (Fig. 2A) then analysed by lectin dot blot (Fig. 2B). The observation of dots was specifically dependent on lectin binding as signal was absent from membranes probed with streptavidin-HRP alone. Untreated lysates showed binding of both lectins, whereas in samples from explants that had been exposed to cerivastatin or pravastatin, reduced binding of ePHA and lPHA was observed. Similarly, the binding of ePHA and lPHA was markedly reduced in lysates from placentas exposed to glycosylation inhibitors.

**Figure 3:**
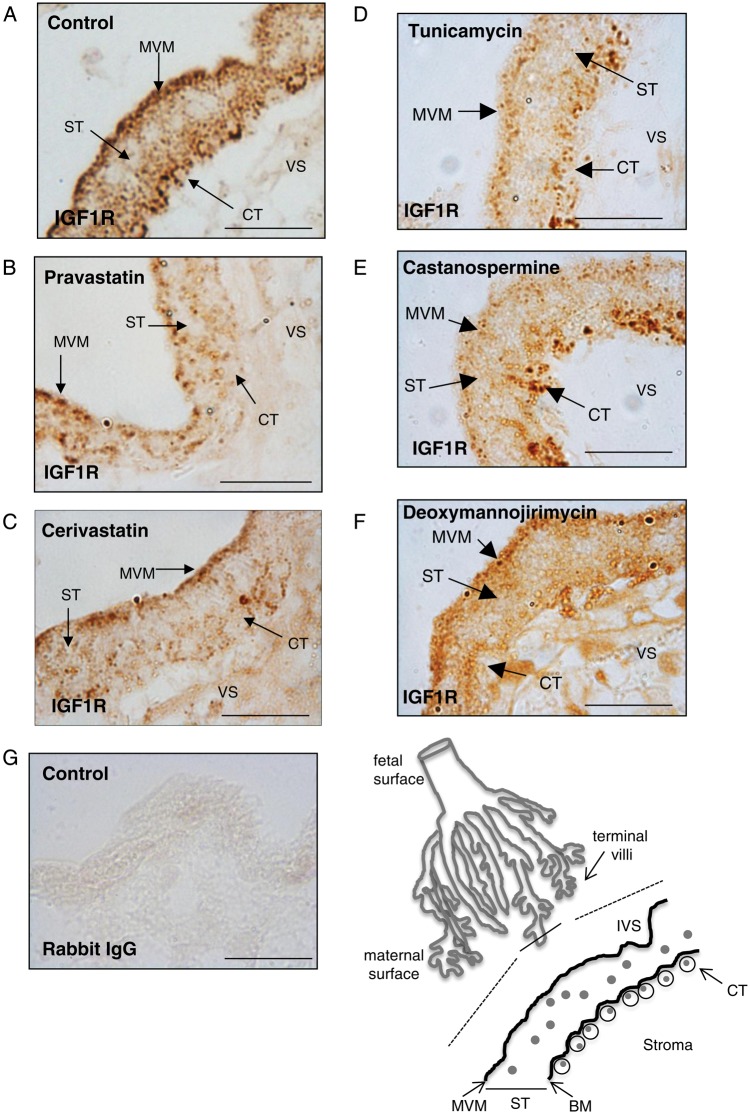
3-Hydroxy-3-methylglutaryl co-enzyme A (HMG-CoA) reductase inhibitors and glycosylation inhibitors alter insulin-like growth factor 1 receptor (IGF1R) distribution in the human placenta. Explants of terminal villi first trimester placenta (dissected from the point shown by the solid line in the line drawing) were untreated (**A**) or incubated in the presence of (**B**) pravastatin (250 nM), (**C**) cerivastatin (50 nM), (**D**) tunicamycin (1 μg/ml), (**E**) castanospermine (5 μg/ml) or (**F**) deoxymannojirimycin (0.5 mM) for 24 h. IGF1R localization was analysed by immunohistochemistry using an IGF1R-specific antibody. In some experiments the primary antibody was replaced by IgG as a negative control (**G**). The intervillous space (IVS), microvillous membrane (MVM), basal membrane (BM), syncytiotrophoblast (ST), cytotrophoblast (CT) and villous stroma (VS) are shown in the line drawing and are indicated by arrows in images (A–F). IGF1R is localized to the MVM, throughout the ST and in CT (A). The expression of IGF1R on the maternal facing MVM and CT plasma membrane was disrupted in the presence of either pravastatin (B) or cerivastatin (C). IGF1R expression on MVM was disrupted by each of the three glycosylation inhibitors. Deoxymannojirimycin also decreased IGF1R expression at the CT cell surface. Each image is representative of staining observed in three individual placentas. Scale bars on images represent 50 μM.

IGF1R is present on the microvillous membrane (MVM), but more prominently in vesicular bodies in the syncytioplasm, especially apically near the MVM, and in cytotrophoblasts (Fig. 3A). Exposure of explants to pravastatin (250 nM; Fig. 3B) or cerivastatin (50 nM; Fig. 3C) disrupted IGF1R distribution; overall the staining declined in intensity and reductions were apparent in the density of stained vesicles in all intracellular locations. Similar changes in distribution were observed following treatment with the three N-glycosylation inhibitors (Fig. 3D–F).

### Supplementation of placental explants with farnesyl pyrophosphate attenuates the effect of HMG-CoA reductase inhibitors on IGF1R localization and IGF-induced cytotrophoblast proliferation

**Figure 4:**
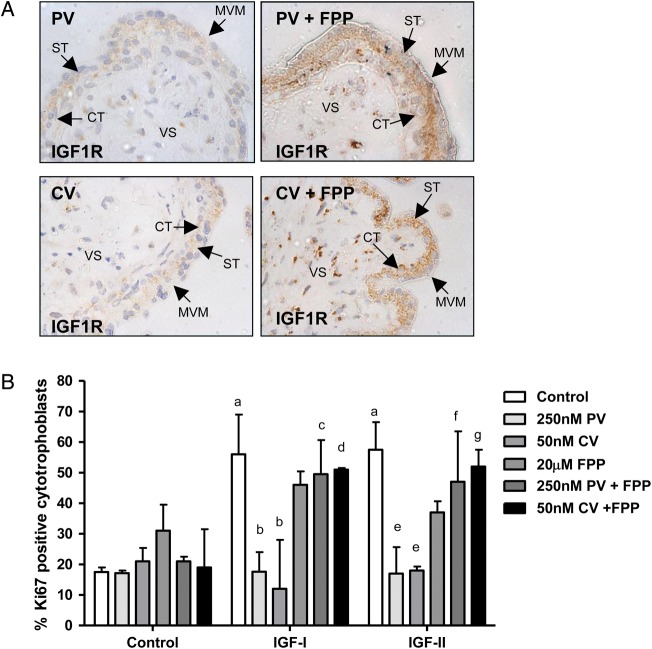
The effect of 3-Hydroxy-3-methylglutaryl co-enzyme A (HMG-CoA) reductase inhibitors on insulin-like growth factor 1 receptor (IGF1R) cell surface expression and IGF-induced proliferation can be attenuated by co-incubation with glycosylation inhibitors. First trimester placental explants (*n* = 5) were incubated in the absence or presence of cerivastatin (CV; 50 nM) or pravastatin (PV; 250 nM) alone or in combination with farensyl pyrophosphate (FPP; 20 μM) for 24 h before the addition of IGF-I (10 nM) or IGF-II (10 nM) and culture for a further 24 h. (**A**) IGF1R localization was analysed by immunohistochemistry using an IGF1R-specific antibody. Arrows indicate microvillous membrane (MVM), syncytiotrophoblast (ST), cytotrophoblast (CT) and villous stroma. Pravastatin and cerivastatin do not disrupt IGF1R expression in the presence of FPP. Each image is representative of staining observed in three individual placentas. Scale bars on images represent 50 μM. (**B**) Proliferation was assessed by using immunohistochemistry to determine the number of Ki67-positive cytotrophoblast as a percentage of total cytotrophoblast and data are presented as median and range of five independent experiments. A Kruskal–Wallis test followed by a Dunn's *post hoc* test was used to assess significance (*P* < 0.05; *n* = 5) a = different from control, untreated tissue; b = different from IGF-I treated tissue; c = different from tissue treated with pravastatin and IGF-I; d = different from tissue treated with cerivastatin and IGF-I ; e = different from IGF-II treated tissue; f = different from tissue treated with pravastatin and IGF-II; g = different from tissue treated with cerivastatin and IGF-II.

Our data suggest that it may be possible to prevent the adverse effects of statins by promoting protein glycosylation. The intermediary metabolite in the conversion of mevalonate to the dolichol phosphate needed for subsequent protein glycosylation is farnesyl pyrophosphate; thus we investigated whether supplementation of placental explants with farnesyl pyrophosphate could prevent statin-mediated disruption of IGF1R cell surface expression and, in turn, IGF-induced proliferation. Figure 4A shows that farnesyl pyrophosphate (20 μM) added to explants at the same time as statin can reduce the disruption to IGF1R cell surface distribution. Moreover, cerivastatin and pravastatin attenuation of IGF-I (10 nM) and -II (10 nM)-induced cytotrophoblast proliferation was overcome by co-incubation of explants with farnesyl pyrophosphate (*P* < 0.05; Fig. 4B).

## Discussion

**Figure 5:**
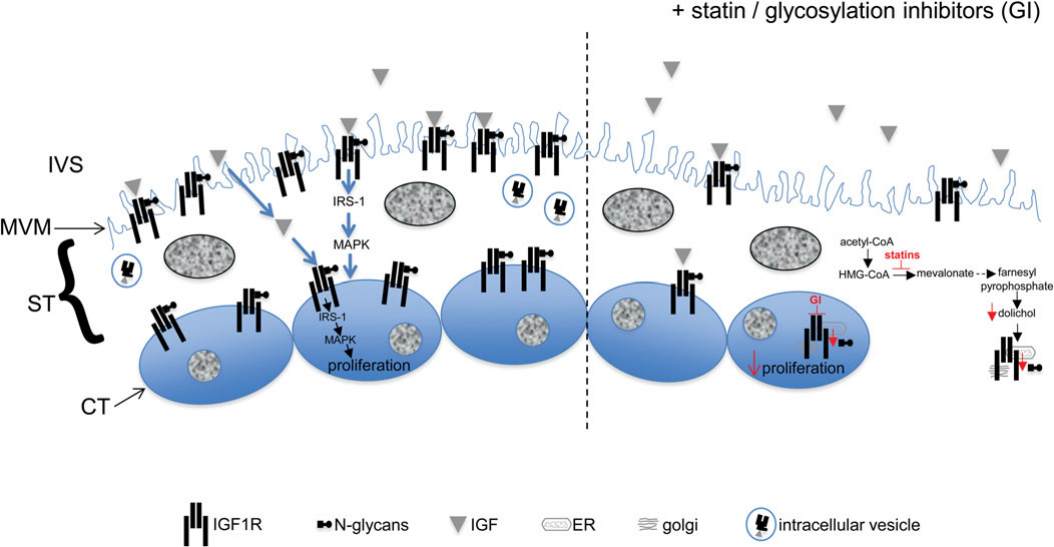
Schematic representation of the effect of statins/glycosylation inhibitors on insulin-like growth factor 1 receptor (IGF1R) and cytotrophoblast proliferation. IGF present in the intervillous space (IVS) stimulates the N-glycosylated IGF1R located at the microvillous membrane (MVM) of syncytiotrophoblast (ST) and/or cytotrophoblast (CT) plasma membrane to activate intracellular signalling molecules, previously shown to include MAPK (Forbes *et al.*, 2008b), and promote cytotrophoblast proliferation. We propose that statins inhibit the production of mevalonate and downstream pathways (represented by dotted arrow) to result in a reduction in the levels of farnesyl pyrophosphate, the precursor of the dolichol phosphate that is needed for N-glycosylation in the ST (as shown) and in the CT. As a consequence, the glycosylation of IGF1R in the endoplasmic reticulum (ER)/golgi apparatus is reduced which leads to less IGF1R being expressed at the MVM/cytotrophoblast cell surface and abolition of IGF-stimulated cytotrophoblast proliferation. Glycosylation inhibitors (GI) also affect IGF1R glyco-processing in the ER/golgi apparatus of ST/CT and thus the functional effects of these compounds are similar to those observed in response to statins.

Previous work has shown that in human placenta, IGF1R exists as multiple glycoforms which bear complex-, hybrid- or high mannose-type N glycans (Masnikosa *et al.*, 2006). In other tissues, glycosylation of IGF1R, in particular, modification of N913 (Kim *et al.*, 2012), is necessary for its presentation at the cell surface (Dricu *et al.*, 1999; Girnita *et al.*, 2000; Itkonen and Mills, 2013) and consequently, its availability for ligand binding (Girnita *et al.*, 2000; Siddals *et al.*, 2004, 2011). Inhibition of HMG-CoA reductase by statins (Stancu and Sima, 2001) results in depletion of mevalonate, and downstream of this, a reduction in the supply of dolichol phosphate needed for N-glycosylation (Carroll *et al.*, 1992); given these effects on cellular metabolism, we hypothesized that our previous finding of reduced trophoblast proliferation in statin-exposed placental explants (Forbes *et al.*, 2008a) was caused by a reduction in IGF1R N-glycosylation. Our current data are consistent with this hypothesis as the glycosylation, localization and function of IGF1R in placental explants treated with either cerivastatin or pravastatin were similar to that observed in tissue cultured with direct inhibitors of glycosylation (Fig. 5). Moreover, these effects could be circumvented by supplying explants with the dolichol phosphate precursor, farnesyl pyrophosphate. The reduction in cell surface receptor abundance seen in response to all treatments is most simply explained by the degradation of misfolded or glucosylated (immature) precursor blocked from release at the ER. The turnover time of IGF1R in placenta is not known, but it is reasonable to speculate that immunoreactive receptor detected after drug exposure represents the remains of the starting pool. Our findings are in keeping with other studies that have investigated the effect of statins on IGF1R processing and signalling in a variety of normal and transformed cell lines (Dricu *et al.*, 1997, 1999; Girnita *et al.*, 2000; Sekine *et al.*, 2008; Siddals *et al.*, 2011). In humans, IGF signalling through IGF1R is a key determinant of cytotrophoblast mitogenesis and placental expansion (Forbes *et al.*, 2008b). However our previous work (Forbes *et al.*, 2008b, 2012) suggests that this pathway is more important for mediating maternally regulated placental growth as cytotrophoblast proliferation *ex vivo* seems to be independent of placentally derived IGF. This is consistent with our current data showing that despite causing a decrease in IGF1R glycosylation, statins do not affect basal proliferation. Work in our own and other labs suggests that EGFR activity contributes to the endogenous regulation of trophoblast proliferation (Forbes and Westwood, 2010; Forbes *et al.*, 2012). EGFR membrane expression, affinity for ligand, dimerization and intracellular signalling partners are all affected by N-glycosylation status (Whitson *et al.*, 2005; Contessa *et al.*, 2008) and there is evidence that statins have a detrimental effect on its activation and function (Liao *et al.*, 2008; Zhao *et al.*, 2010). However, deglycosylated EGFR does retain some signalling activity (Soderquist and Carpenter, 1984; Wang *et al.*, 2001).

Clinical trials are currently testing statins, in particular pravastatin, for improving placental vascular function in pregnancy complicated by pre-eclampsia (ISRCTN, 2009; Costantine and Cleary, 2013). *In vivo* studies support the notion that statins might have a positive effect on vascular function in placenta, as pravastatin treatment of two different mouse models of pre-eclampsia caused an improvement in vascular reactivity (Costantine *et al.*, 2010), placental vasculogenesis (Cantoni *et al.*, 2011) and blood flow (Singh *et al.*, 2011). All of these studies administered pravastatin at embryonic day 7.5+ which, given our data on human primary trophoblast, would be predicted to affect placental growth. The fact that none of the studies have reported reduced placental or fetal weights may be explained by inter-species differences in the role of IGF1R. In humans altered IGF1R expression or function is associated with aberrant placental and fetal growth (Walenkamp *et al.*, 2006; Kruis *et al.*, 2010) whereas in mice, the role of IGF1R in the placenta is less clear. The birthweight of both *Igf1* and *Igf1r* null mice is reduced without changes to placental growth (Baker *et al.*, 1993). Furthermore, whilst the reduced fetal growth observed in *Igf2* knockout mice can be attributed to aberrant placental development and function (Baker *et al.*, 1993; Constancia *et al.*, 2002, 2005; Sibley *et al.*, 2004), the finding that *Igf2* and *Igf-2/Igf1r* double mutants have an identical placental phenotype suggests that IGF-II does not signal through IGF1R in the placenta (Baker *et al.*, 1993). Therefore, in the murine placenta, statin-mediated alteration of IGF1R glycosylation may not affect trophoblast proliferation.

Further evidence for species-dependent effects of statins in pregnancy comes from studies investigating miscarriage and pre-term labour (PTL). Both simvastatin and pravastatin prevent fetal loss in a mouse model of anti-phospholipid syndrome (Girardi, 2009) but pravastatin was ineffective in a human trophoblast cell line model of this condition (Odiari *et al.*, 2012). Similarly, statins protected against PTL induced by lipopolysaccharide administration (Gonzalez *et al.*, 2014) yet some studies report that in women, PTL is more frequent in those exposed to statins in early gestation (Winterfeld *et al.*, 2013).

Recent studies using the dually perfused human placenta have shown that pravastatin can cross the placenta (Nanovskaya *et al.*, 2013; Zarek *et al.*, 2013) and thus it is possible that in addition to indirectly affecting pregnancy outcome by influencing placental growth/function, statins may have a direct effect on fetal development. Indeed, the FDA have assigned statins to pregnancy category X (Girardi, 2013) although the latest systematic review and meta-analysis of available data suggest that they are unlikely to be teratogenic (Kusters *et al.*, 2012). Our data, and that of others using models of human placenta (Kenis *et al.*, 2005; Tartakover-Matalon *et al.*, 2007; Odiari *et al.*, 2012), suggest that due to their effect on early placental development, women should avoid using statins during the first trimester; the outcomes of the trials that are administering statins at later time points will be useful in exploring their potential as a therapy for complications manifesting in late pregnancy.

## Authors' roles

K.F., K.S., M.W. and J.D.A. and designed the research study; K.F. and V.K.S. performed the research; K.F., K.S., M.W. and J.D.A. analysed the data; M.G. provided essential reagents; K.F., M.W. and J.D.A. wrote the paper; K.S. and M.G. critically reviewed the paper.

## Funding

This work was supported by the Biotechnology and Biological Sciences Research Council (grant number BB/E007678/1); a University of Manchester Stepping Stones Fellowship (to K.F.); and a Wellcome Trust Summer Vacation Studentship (to V.K.S.). The Maternal and Fetal Health Research Centre is supported by funding from Tommy's the Baby Charity, an Action Research Endowment Fund, the Manchester Biomedical Research Centre and the Greater Manchester Comprehensive Local Research Network. Funding to pay the Open Access publication charges for this article was provided by the Biotechnology and Biological Sciences Research Council.

## Conflict of interest

None declared.
